# Transcription factor Tcf4 is the preferred heterodimerization partner for Olig2 in oligodendrocytes and required for differentiation

**DOI:** 10.1093/nar/gkaa218

**Published:** 2020-04-08

**Authors:** Miriam Wedel, Franziska Fröb, Olga Elsesser, Marie-Theres Wittmann, D Chichung Lie, André Reis, Michael Wegner

**Affiliations:** 1 Institut für Biochemie, Emil-Fischer-Zentrum, Friedrich-Alexander-Universität Erlangen-Nürnberg, Erlangen, Germany; 2 Humangenetisches Institut, Universitätsklinikum Erlangen, Friedrich-Alexander-Universität Erlangen-Nürnberg, Erlangen, Germany

## Abstract

Development of oligodendrocytes and myelin formation in the vertebrate central nervous system is under control of several basic helix-loop-helix transcription factors such as Olig2, Ascl1, Hes5 and the Id proteins. The class I basic helix-loop-helix proteins Tcf3, Tcf4 and Tcf12 represent potential heterodimerization partners and functional modulators for all, but have not been investigated in oligodendrocytes so far. Using mouse mutants, organotypic slice and primary cell cultures we here show that Tcf4 is required in a cell-autonomous manner for proper terminal differentiation and myelination *in vivo* and *ex vivo*. Partial compensation is provided by the paralogous Tcf3, but not Tcf12. On the mechanistic level Tcf4 was identified as the preferred heterodimerization partner of the central regulator of oligodendrocyte development Olig2. Both genetic studies in the mouse as well as functional studies on enhancer regions of myelin genes confirmed the relevance of this physical interaction for oligodendrocyte differentiation. Considering that alterations in TCF4 are associated with syndromic and non-syndromic forms of intellectual disability, schizophrenia and autism in humans, our findings point to the possibility of an oligodendroglial contribution to these disorders.

## INTRODUCTION

Oligodendrocytes represent one of the main cell types in the vertebrate central nervous system (CNS). Their task is to enwrap axons with myelin sheaths to permit saltatory conduction and provide trophic support. Oligodendrocytes are of neuroectodermal origin and develop from neuroepithelial progenitor cells of the ventricular zone in an ordered series of events. This includes specification to oligodendrocyte precursor cells (OPCs), and expansion of the OPC population, followed by progression to pre-myelinating and eventually myelinating oligodendrocytes during terminal differentiation (1). Many of these steps are regulated on the transcriptional level by a complex network that contains several basic helix-loop-helix (bHLH) transcription factors as central components (2,3).

The family of bHLH proteins is subdivided into seven classes (4). Members of classes II, V and VI have so far been shown to influence oligodendrocyte development (5). This includes the class II bHLH protein Olig2, which is expressed throughout oligodendroglial development and required from specification to terminal differentiation (6,7). Other class II bHLH proteins with roles in oligodendroglial development include Olig1 as a transcription factor predominantly active during terminal differentiation and Ascl1 (also known as Mash1) as a factor required early for the generation of OPCs and again late in differentiating oligodendrocytes (6,8,9). Additionally, the class V proteins Id2 and Id4 as well as the class VI protein Hes5 influence oligodendroglial development as transcription factors that maintain OPCs in the proliferative state and counteract differentiation (10–12).

Class II bHLH proteins are potent regulators of cell type specification and differentiation in numerous developmental processes. For full functionality, efficient binding to DNA must be ensured and this usually requires heterodimerization with class I bHLH proteins (13,14). Class I bHLH proteins also heterodimerize with class V and class VI factors and influence their activities (15,16). In vertebrates, they consist of the three paralogs Tcf3 (NCBI GeneID: 21423, also known as E2A, E12 or E47), Tcf4 (NCBI GeneID: 21413, also known as E2-2, Itf2 or Sef2) and Tcf12 (NCBI GeneID: 21406, also known as bHLHb20 or HEB/REB). Considering the impact of class I bHLH proteins on overall bHLH function it is surprising that this class of bHLH proteins has not yet been thoroughly studied in the oligodendrocyte lineage.

**Figure 1.**
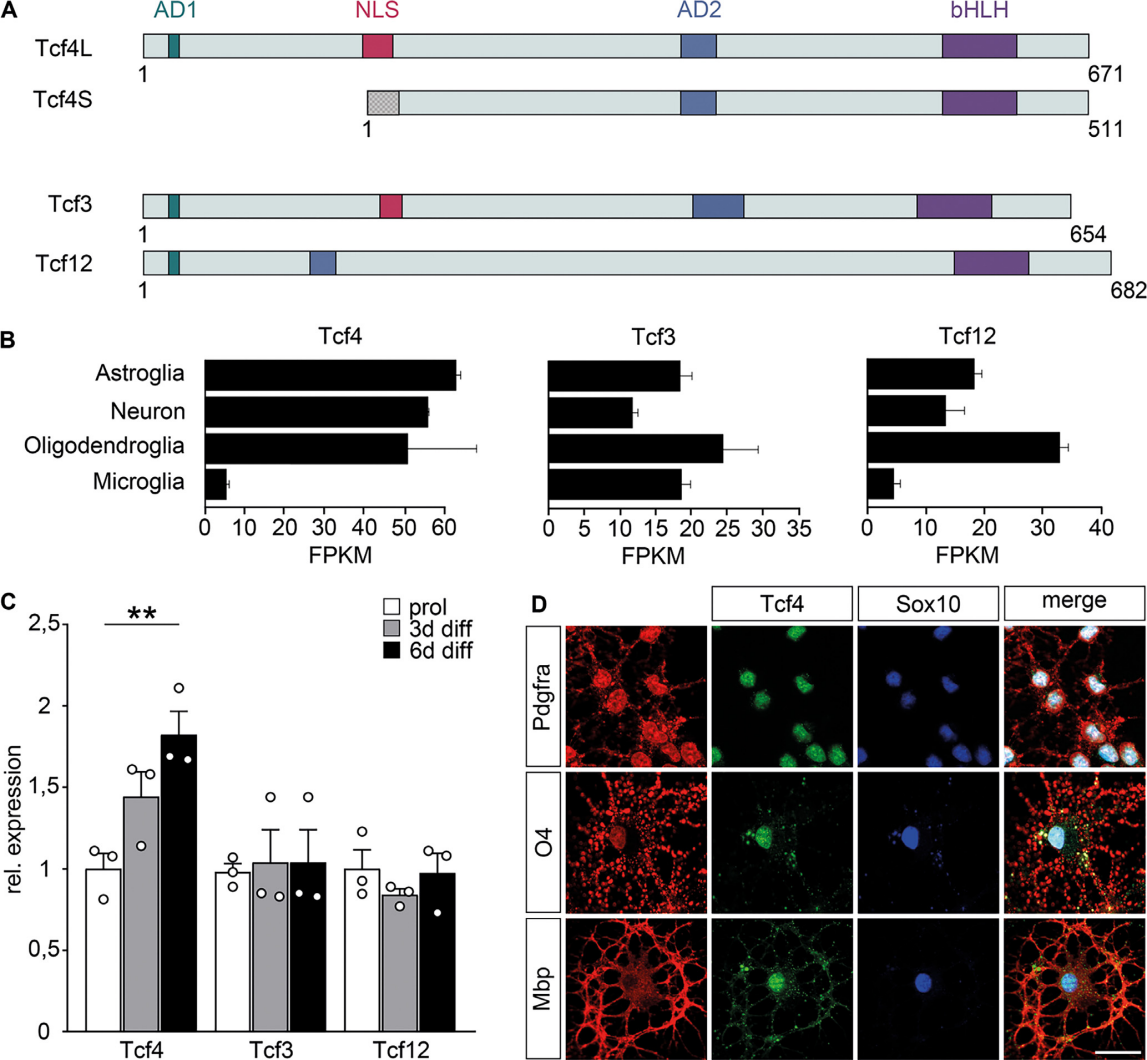
Class I bHLH expression in oligodendroglial cells. (**A**) Schematic representation of class I bHLH proteins. For Tcf4, both long (Tcf4L) and short (Tcf4S) isoforms are shown. Colored boxes correspond to nuclear localization signal (NLS, red), transactivation domains (AD1, AD2, blue) and the DNA-binding basic helix-loop-helix domain (bHLH, pink). The checkered box at the N-terminus of Tcf4S corresponds to a region unique to Tcf4S in comparison to Tcf4L. Numbers on the right of each protein indicate length in amino acid residues. (**B**) Expression of class I bHLH factors in various CNS cell types according to GSE52564 (40). (**C**) Quantitative RT-PCR to determine Tcf3, Tcf4 and Tcf12 transcript levels in cultured oligodendroglial cells kept in proliferating conditions or placed under differentiating conditions for 3 or 6 days. Transcript levels for each gene under proliferating conditions were set to 1 and levels under differentiating conditions were set in relation to this (n = 3). Differences to controls were statistically significant as determined by two-tailed Student's *t* test (**P ≤ 0.01). (**D**) Co-immunocytochemistry on cultured oligodendroglial cells using antibodies directed against Tcf4 (green) in combination with antibodies directed against Sox10 (blue) as well as Pdgfra, O4 and Mbp (all in red). A merge is shown in addition to single channels. Scale bar: 25 μm.

To make inroads into this important topic, we analysed the role of Tcf4 in oligodendroglial development. The *Tcf4* gene is transcribed from two alternative promoters. As a consequence, Tcf4 proteins exist as long (Tcf4L) and short (Tcf4S) isoforms (Figure 1A). Only Tcf4L contains a nuclear localization signal and AD1, the aminoterminal of two transactivation domains potentially present in Tcf4 proteins. As a consequence, Tcf4L exhibits a stronger nuclear enrichment and a higher transcriptional activity than Tcf4S (17,18). In the immune system, Tcf4L has been shown to be selectively expressed in plasmacytoid as opposed to classical dendritic cells and to be essential for their specification (19). Although this argues for isoform-specific developmental functions, this topic has not been thoroughly analysed so far.

In humans, inactivating mutations in or loss of *TCF4* cause Pitt-Hopkins syndrome in the heterozygous state (20,21). Pitt-Hopkins syndrome is a severe form of intellectual disability associated with other symptoms including breathing abnormalities, epileptic seizures and altered facial morphology. *TCF4* deletions have also been detected in patients with autism-spectrum disorders and common gene variants appear associated with an increased risk of schizophrenia (22). Given its requirement for many aspects of CNS development, we hypothesized that Tcf4 may also have important roles in oligodendroglial cells and CNS myelination.

It deserves to be mentioned that this class I bHLH protein is unrelated to Tcf7l2 (NCBI GeneID: 21416), a HMG-domain containing transcription factor and nuclear effector of canonical Wnt signalling that has been extensively studied in oligodendrocytes under its alternative name Tcf4 (23,24).

Here, we show that the class I bHLH protein Tcf4 is required for proper terminal differentiation in the oligodendrocyte lineage. We furthermore provide genetic and biochemical evidence that Olig2 predominantly interacts with Tcf4 among class I bHLH proteins and propose a model, in which the preferential partnership with Olig2 and their joint action on enhancers of myelin genes explain at least in part the Tcf4 requirement during oligodendrocyte differentiation.

## MATERIALS AND METHODS

### Mice and organotypic slice cultures

Mice carrying the *Tcf4^tm1a(EUCOMM)^^Wtsi^* allele (generated by the Mouse Knockout Project) were interbred or crossed to mice carrying the *Olig2^tm1(^^cre^^)^^Tmj^* (25) or the *Sox10^lacZ^* (26) allele to obtain embryos with homozygous deletion of the long isoform of Tcf4 (Tcf4ko) or embryos and pups with combined heterozygous loss of Olig2 and Tcf4L (OT double het) or Sox10 and Tcf4L (ST double het). Genotyping was according to published protocols. All mice were on a mixed C3H × C57Bl/6J background. They were kept under standard housing conditions with 12:12 hours light-dark cycles and continuous access to food and water in accordance with animal welfare laws. Experiments were approved by the responsible local committees and government bodies. Both male and female embryos and pups were used for the study. Embryos were obtained at embryonic day (E) 14.5, 16.5 or 18.5, pups at birth or postnatal day 7.

For organotypic slice culture, 300 μm thick coronal slices were generated from forebrains of newborn control and Tcf4ko mice at the level of the corpus callosum using a Leica VT1000S vibratome. Slices were placed on 0.4 μm Millicell-CM™ organotypic cell culture inserts (Merck-Millipore) and incubated for up to 28 days in medium containing 25% heat-inactivated horse serum (Gibco). Slice culture medium was changed every second day.

### Tissue preparation, immunohistochemistry and *in situ* hybridization

For immunohistochemistry, tissues from embryos or pups underwent fixation in 4% paraformaldehyde, cryoprotection in 30% sucrose, embedding and freezing at -80°C before sectioning on a cryotome at 10 μm thickness. Cultured forebrain slices were used after fixation in 4% paraformaldehyde and permeabilization in 0.5% Triton X-100.

The following primary antibodies were applied: guinea pig anti-Sox10 antiserum (1:1000 dilution, (27)), rabbit anti-Myrf antiserum (1:1000 dilution, (28)), rabbit anti-Pdgfra antiserum (1:300 dilution, Santa Cruz, #sc-338, Lot# E-1210), rabbit anti-Olig2 antiserum (1:1000 dilution, Millipore #AB9610, Lot# 2060464), rabbit anti-Ki67 antiserum (1:500 dilution, Thermo Fisher Scientific, #RM-9106, Lot#9106S906D), rabbit anti-cleaved caspase 3 antiserum (1:200 dilution, Cell Signaling Technology, #9661, Lot#0043) and mouse anti-Nefm monoclonals (1:200 dilution, Iowa Developmental Studies Hybridoma bank). Secondary antibodies were coupled to Cy3 (Dianova) or Alexa488 (Molecular Probes) fluorescent dyes. Samples were documented with a Leica DMI 6000B inverted microscope (Leica) equipped with a DFC 360FX camera (Leica) as well as a Zeiss LSM 780 confocal microscope.

For *in situ* hybridization, 10 μm cryotome sections from spinal cord were incubated with DIG-labeled antisense riboprobes specific for *Mbp* and *Plp1* as described (29). Samples were analysed and documented with a Leica MZFLIII stereomicroscope equipped with an Axiocam (Zeiss).

For determination of colocalized signals on cultured slices images were processed with the ImageJ Colocalization Threshold tool. Colocalization was measured as correlation of pixel intensities over all pixels in the image. Standard settings were used.

### Cell culture, transductions and transfections

Both primary cells and cell lines were used. Cell lines included human embryonic kidney 293 cells (obtained from ATCC) and mouse N2a neuroblastoma (obtained from ATCC). N2a cells, but not Hek293 cells were authenticated by PCR. Hek293 cells were used for extract preparation after transfection with polyethylenimine (30) or virus production after transfection with Lipofectamine 2000 (Thermo Fischer Scientific). Cell lines were grown in DMEM supplemented with 10% fetal calf serum (FCS). N2a cells were transfected with Superfect reagent (Qiagen) and used for luciferase assays (31).

Rodent primary oligodendroglial cells were obtained from brain tissue of newborn mice or Wistar rats of both sexes. Mouse and rat OPC cultures were prepared from mixed glial cultures by shake-off (32). For proliferation, OPCs were grown on poly-ornithine in defined medium containing 10 ng/ml PDGF-AA and 10 ng/ml Fgf2. OPCs were transduced with retroviruses at a MOI of 0.5–1 or transfected with expression plasmids using Xfect (TaKaRa). For differentiation, growth factors were replaced by 40 ng/ml T3 and 0.5% FCS.

### Immunocytochemistry

Immunocytochemistry was performed essentially as described and involved cell fixation in 4% paraformaldehyde, permeabilization (except for O4 staining), blocking and consecutive incubation with primary and secondary antibodies, separated by extensive washing cycles (33). In addition to antibodies already mentioned for their use in immunohistochemistry, the following primary antibodies were used: mouse anti-O4 monoclonal (R&D systems, #MAB1326, Lot# HWW1115081), rabbit anti-Tcf4 (Abcam, #ab185736, Lot#GR3229215-1), rabbit anti-GFP antiserum (Molecular Probes, #A11122, Lot# 1293114), rat anti-MBP monoclonal (Abcam, #ab7349, Lot# GR188102-12 and Bio-Rad, #MCA409S, Lot #210610). Secondary antibodies were coupled to Cy3 (Dianova) or Alexa-Fluor (Molecular Probes) fluorescent dyes. Stainings were documented on a Leica DMI 6000B inverted microscope or a Zeiss Apotome.

### Quantitative RT-PCR

RNA was prepared from mouse spinal cord and rat oligodendroglial cultures, reverse transcribed and used to analyse expression levels by quantitative PCR on a Biorad CFX96 Real Time PCR System. The following primer pairs were used: 5′-GGGATCATGCGCACAGTCTA-3′ and 5′-AAGTGCCGATTCCACCTCTG-3′ for *Acss2*, 5′-TCTGTCTCCTCTCTCCCTGC-3′ and 5′-CCCTTCTTGGCTTCAGGAGG-3′ for *Fa2h*, 5′-CCCAAGTACCGTGGCGGTGG-3′ and 5′-GCGGCGAAGGCTTTGCTGTG-3′ for *Hes5*, 5′-CTTGCCTGAGGATCCGTGTT-3′ and 5′-GCAGAGCTCGGTACTGTCTC-3′ for *Hsd17b7*, 5′-CCCGGTGGACGACCCGATG-3′ and 5′-CAGATGCCTGCAAGGACAGGATGC-3′ for *Id2*, 5′-GAGACTCACCCTGCTTTGCT-3′ and 5′-CTGTCACCCTGCTTGTTCAC-3′ for *Id4*, 5′-TGCCTTTCTCTCTGGCCATG-3′ and 5′-AGGGGAATGGGGATCAGACA-3′ for *Lss*, 5′-GAGTGGGGGCTACTGGTTTC-3′ and 5′-AGACAAAAGGGAGCCTCTGC-3′ for *Mboat1*, 5′-CCTGTGTCCGTGGTACTGTG-3′ and 5′-TCACACAGGCGGTAGAAGTG-3′ for *Myrf*, 5′-GCTGAAGAACCTGTGGGTGT-3′ and 5′-GGTACTCCTTCTGGGGTGGT-3′ for *Nefm*, 5′-AGCACGATGACTCTGAGACC-3′ and 5′-TTGGCTACGTGAAGATAGGG-3′ for *Cspg4*, 5′-GAAGCAGATGACTGAGCCCGAG-3′ and 5′-CCCGTAGATCTGCTCACCAG-3′ for *Olig2*, 5′-GGAGAACCTGTTGCCGGGAC-3′ and 5′-TCTCGATGGCACTCTCTTCC-3′ for *Pdgfra*, 5′-CTCCCTTCCTCGGTGTATCC-3′ and 5′-CTAGGCGTACTCCAGAGCTC-3′ for *Tcf3*, 5′-ACTACTATAATGGGAAAGCGGTC-3′ and 5′-CATGAAGAAGGAGCTAGGGAAAG-3′ for *Tcf4* short isoform, 5′-ACGGACAAAGAGCTGAGTGA-3′ and 5′-CCCTGCTAGTCATGTGGTCA-3′ for *Tcf4* long isoform, 5′-GACCACACGAACAACAGCTT-3′ and 5′-TCTTCGATTCGGCTTTGCAG-3′ for total *Tcf4* and 5′-ACCAGCAGCTCACCATATGT-3′ and 5′-TGCTGTGAGAGGTGAAGGAG-3′ for *Tcf12*. Primer pairs for *Ank2*,*Gapdh, Gjc2, Mag, Mbp*, *Nfasc, Nkx2.2, Plp1, Rpl8, Rplp0* and *Sox10*, were as described (34–36). Transcript levels were normalized to *Gapdh, Rpl8* and *Rplp0*.

### Chromatin immunoprecipitation

Chromatin was prepared from rat oligodendroglial cultures kept under differentiating conditions after treatment with 1% paraformaldehyde and shearing to 200–400 bp fragments in a Bioruptor (Diagenode) (37). After pre-clearing, chromatin was incubated with rabbit antisera against Tcf4 or Sox10, mouse monoclonals against Olig2 (Millipore, #MABN50, Lot# 2730966), rabbit preimmune antiserum or mouse IgGs before addition of protein A sepharose beads and precipitation. After crosslink reversal, proteinase K treatment and purification of DNA from input and precipitated chromatin, the following primer pairs were used in quantitative PCRs on a Bio-Rad CFX96 thermocycler with each reaction performed in triplicates: 5′-CACGATGACACTGTTGACCA-3′ and 5′-CACTTGTTCAATGCCTGTGG-3′ (amplifying Chr15:36146843–36146945 according to rn4, near *Gjb2*), 5′-CTCTCCCTTTGTCAGCTGGA-3′ and 5′-CAGTGTGAATCTGGGAACTCA-3′ amplifying ChrX:124493220–124493330 according to rn4, within *Plp1*) and 5′-TCCAGATGTGAGAGAAAAACAA-3′ and 5′-CACCGAGTACAGAAAAGGTCCA-3′ amplifying Chr9: 110871476–110871422 according to rn4 as control region). The ΔΔCt method was used to calculate the recovery rates of a given DNA segment relative to the total input. Recovery rates for a specific antiserum were compared to recovery with preimmune antiserum, those for monoclonals to IgGs. Recovery rates for preimmune serum and IgGs were arbitrarily set to 1.

### Plasmids and retroviruses

Expression plasmids for Sox10, Olig2 (NM_016967), Olig1 (NM_021770.4), Ascl1 (NM_008553), Tcf3 (NM_011548), Tcf4L (NM_001369568), Tcf4S (NM_001243235.2) and Tcf12 (NM_013176.2) were based on pCMV5. Those for Sox10 and Olig2 have been described before (30,38). Expression plasmids for Tcf3, Tcf4L and Tcf12 contained the corresponding coding sequences inserted in frame behind a N-terminal myc-tag, wheras expression plasmids for Olig1 and Ascl1 contained the corresponding coding sequences in frame with a N-terminal T7-tag. Coding sequences for Tcf3, Tcf4L and Tcf12 were additionally inserted into pCAG–IRES–GFP (39) to produce retroviruses for ectopic expression of these class I bHLH proteins.

ECRs were localized on Chr15:36146837–36147317 for the *Gjb2* ECR and on ChrX:124492788–124493689 for the *Plp1* ECR according to rn4, amplified by PCR from genomic DNA and inserted into the pGL2 luciferase reporter plasmid in front of a β-globin minimal promoter.

### Extract preparation, co-immunoprecipitation and luciferase assays

For co-immunprecipitation experiments, whole cell extracts were prepared by lysing transfected Hek293 cells in 10 mM HEPES pH 7.9, 10 mM KCl, 0.1 mM EDTA, 0.1 mM EGTA. After addition of NP-40 to 1% final concentration and NaCl to 400 mM, 15 min rotation at 4°C and 5 min centrifugation, glycerol was added to the supernatant at a final concentration of 10% (37).

For co-immunoprecipitation extracts were incubated either with mouse anti-myc monoclonal antibody (Cell Signaling, #2276, Lot#0024) mouse anti-T7 monoclonal antibody (Novagen, #69552, Lot#2982701) or rabbit anti-Olig2 antiserum and protein A sepharose beads (GE Healthcare). After extensive washing, bead-bound material was eluted by boiling in 150 mM Tris–HCl, 6% SDS, 15% β-mercaptoethanol, 30% glycerine, 0.3% bromophenol blue and analysed after size separation on 10% SDS-polyacrylamide gels by western blotting. The following primary antibodies and detection reagents were used: rabbit anti-Olig2 antiserum (1:1000 dilution), mouse anti-myc monoclonal (1:10 000 dilution), mouse anti-T7 monoclonal (1:1000 dilution) and horseradish peroxidase coupled to protein A (Zymed, #10-1023, Lot#20873065, 1:3000 dilution). Detection was by chemiluminescence using ECL reagent. Western blots were densitometrically scanned and have been cropped for presentation. Band intensities were measured with NIH ImageJ using standard parameters.

For luciferase assays in N2a cells, 0.5 μg of pCMV5-based expression plasmid and 0.5 μg of luciferase reporter were used per well of a 24-well plate. Each single transcription factor expression plasmid was supplied at 0.167 μg. Overall amounts of plasmid in a particular experiment were kept constant by adding empty pCMV5 where necessary. Whole cell extracts were prepared 48 h post transfection and luciferase activities were determined in the presence of luciferin substrate by detection of chemiluminescence.

### Statistical analysis

To determine whether differences in cell numbers, transcript levels or luciferase activities were statistically significant, a two-tailed Student's *t* test or one way ANOVA with Bonferroni correction was performed as appropriate (*P ≤ 0.05; **P ≤ 0.01, ***P ≤ 0.001). Results from independent animals or transfections were treated as biological replicates (*n* ≥ 3).

## RESULTS

### Class I bHLH expression in oligodendroglial cells

Tcf3, Tcf4 and Tcf12 are the three class I bHLH proteins (Figure 1A). To monitor their expression in oligodendroglial cells, we analysed publicly available databases (40) and found that all three are expressed in substantial and comparable amounts in oligodendroglial cells at least on transcript level (Figure 1B). Amounts appear furthermore similar to those detected in astrocytes and neurons.

Quantitative RT-PCR confirmed that transcripts for all three class I bHLH proteins are present at substantial levels in oligodendroglial cultures under proliferating and differentiating conditions (Figure 1C). *Tcf4* levels increased slightly during differentiation (82 ± 14% after 6 days), whereas *Tcf3* and *Tcf12* levels remained constant. In case of Tcf4, presence was confirmed on the protein level in Pdgfra-positive OPCs, O4-positive pre-myelinating and Mbp-positive myelinating oligodendrocytes by immunocytochemical staining of cultured rat oligodendroglial cells (Figure 1D). Immunoreactivity was found in both nuclei and cytoplasm.

### Effects of Tcf4 deletion on oligodendroglial development *in vivo*

**Figure 2.**
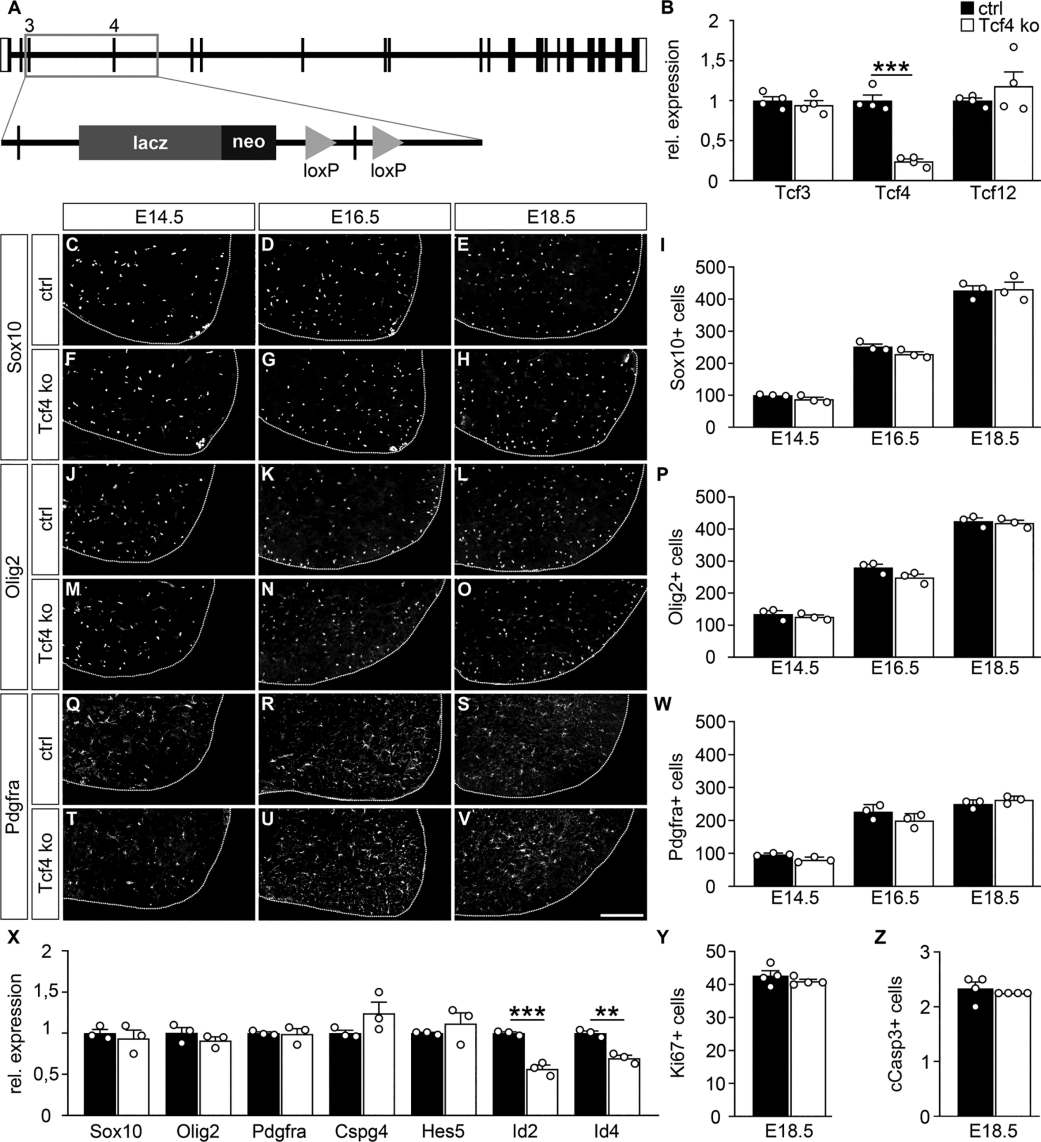
Oligodendroglial development in the prenatal spinal cord of Tcf4ko mice. (**A**) Schematic representation of the mutant *Tcf4* allele with an insertion of a *lacZ* cassette in intron 3 and a floxed exon 4. (**B**) Quantitative RT-PCR to determine *Tcf3, Tcf4* and *Tcf12* transcript levels in the spinal cord of control (ctrl, black bars) and Tcf4ko (white bars) mice at E18.5. Values for each gene in control spinal cord were set to 1 and levels in Tcf4ko spinal cord were set in relation to this (*n* = 4). (**C–W**) Immunohistochemical stainings of spinal cord sections from control (C–E, J–L, Q–S) and Tcf4ko (F–H, M–O, T–V) mice at E14.5 (C, F, J, M, Q, T), E16.5 (D, G, K, N, R, U) and E18.5 (E, H, L, O, S, V) with antibodies directed against Sox10 (C–E, F–H), Olig2 (J–L, M–O) and Pdgfra (Q–S, T–V), and corresponding quantifications (I, P, W). For stainings, the right ventral horn is shown, placed on a black background and demarcated by a stippled line. Scale bar: 100 μm. For quantifications absolute mean numbers of marker-positive cells ± SEM per thoracic spinal cord section of control and Tcf4ko mice are shown (*n* = 3 mice for each genotype, counting three separate sections). (**X**) Quantitative RT-PCR to determine *Sox10*, *Olig2*, *Pdgfra, Cspg4, Hes5, Id2* and *Id4* transcript levels in the spinal cord of control and Tcf4ko mice at E18.5. Values for each gene in control spinal cord were set to 1 and levels in Tcf4ko spinal cord were set in relation to this (*n* = 3). (**Y**, **Z**) Quantification of Ki67-positive (Y) and cleaved caspase 3-positive (Z) oligodendroglial cells in spinal cord sections from control and Tcf4ko mice at E18.5 (*n* = 4 mice for each genotype, counting three separate sections). Differences to controls were statistically significant as determined by two-tailed Student's t test (**P ≤ 0.01; ***P ≤ 0.001).

To study the role of Tcf4 in oligodendroglial development we used a mouse in which insertion of a *lacZ* cassette into intron 3 of the *Tcf4* gene prevents expression of the long isoform Tcf4L (Figure 2A) (19). These mice die within a few hours after birth and exhibit phenotypic defects similar to those reported for mice in which both short and long isoforms were missing (41), indicating that the long isoform is functionally dominant.

Because of the immediate postnatal death, we had to restrict our analysis to the prenatal period. We furthermore focused on the thoracic spinal cord as a region in which at least some of the oligodendroglial cells have begun to differentiate at birth. Quantitative RT-PCR on spinal cord tissue confirmed a strong reduction of *Tcf4* levels in the mutant (Figure 2B). *Tcf3* and *Tcf12* levels remained comparable to those in control spinal cord.

To investigate potential effects on oligodendroglial cells, we first performed immunohistochemical stainings on transverse spinal cord sections of control and Tcf4ko mice for Sox10 as a pan-oligodendroglial marker (30). By visual inspection, the number of Sox10-positive cells and their distribution throughout the spinal cord appeared comparable between age-matched control and Tcf4ko mice at E14.5, E16.5 and E18.5 (Figure 2C–H). Quantification of cell numbers confirmed this impression (Figure 2I). A similar result was obtained when Olig2 was used as a marker of oligodendroglial cells (Figure 2J–P) (6,7). During the late phase of fetal development, most oligodendroglial cells are still in the OPC stage under control conditions. Using Pdgfra as an OPC marker, it became obvious that the majority of oligodendroglial cells in the spinal cord of Tcf4ko mice from E14.5 to E18.5 were also OPCs and that their number closely corresponded to those in the control (Figure 2Q–W). Quantitative RT-PCR experiments on spinal cord at E18.5 confirmed that transcript levels for *Sox10*, *Olig2* and *Pdgfra* were comparable between control and Tcf4ko mice (Figure 2X). Additionally, we failed to detect statistically significant alterations in the amounts of *Cspg4* and *Hes5* transcripts as additional OPC markers (Figure 2X). *Id2* and *Id4* levels were downregulated. However, this downregulation was not reproducible in purified oligodendroglial cells (see Figure 6A). Numbers of Ki67-positive and cleaved caspase 3-positive oligodendroglial cells in Tcf4ko spinal cords furthermore resembled those in the control at E18.5 (Figure 2Y, Z) We conclude from these findings that overall oligodendroglial numbers, proliferation and apoptosis rates as well as their developmental status as OPCs are unaffected in Tcf4ko mice. Tcf4 thus appears dispensable at early stages of oligodendroglial development.

**Figure 3.**
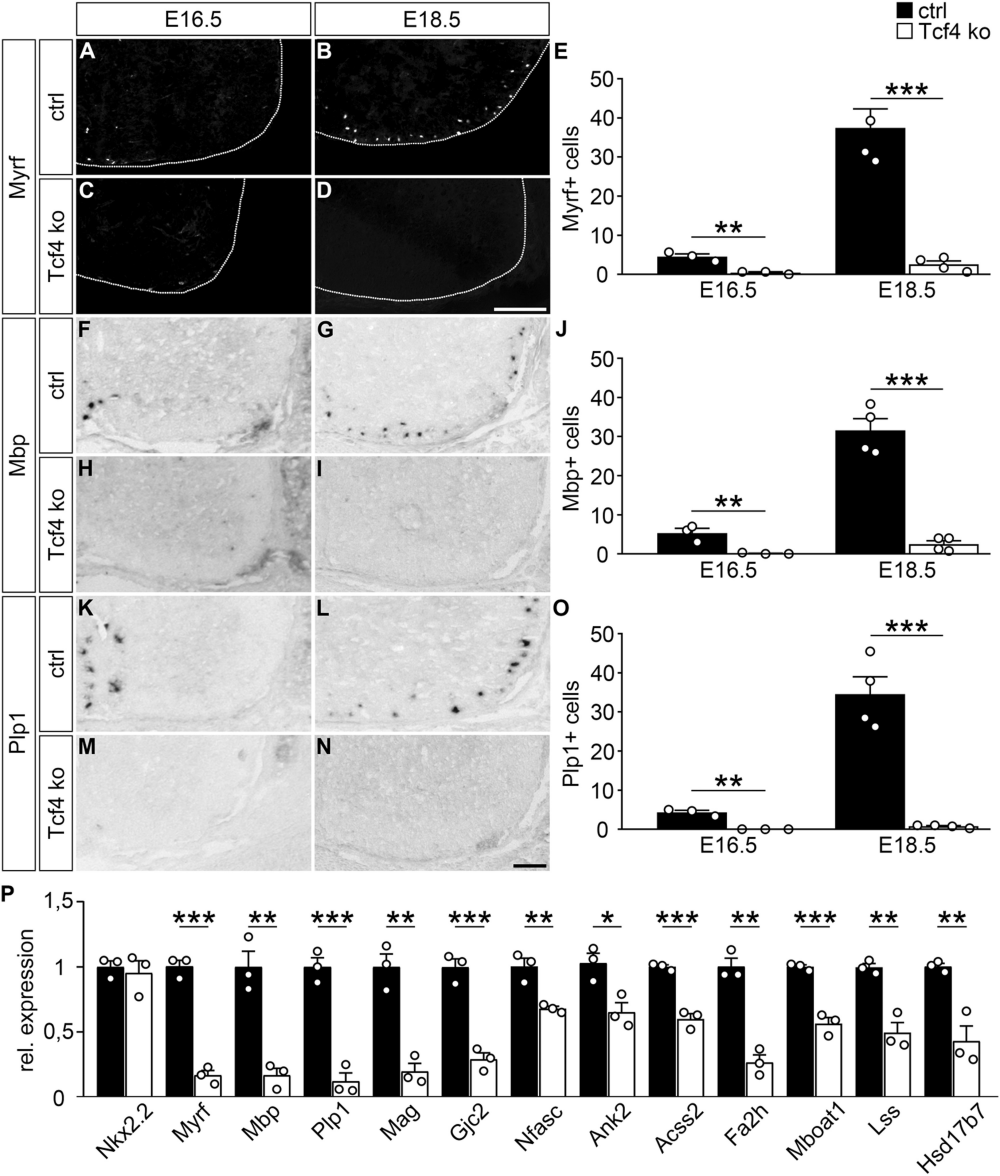
Impaired oligodendroglial differentiation in Tcf4ko mice. (**A–E**) Immunohistochemical stainings of spinal cord sections from control (ctrl) (A, B) and Tcf4ko (C, D) mice at E16.5 (A, C) and E18.5 (B, D) with antibodies directed against Myrf, and corresponding quantifications (E). For stainings, the right ventral horn is shown, placed on a black background and demarcated by a stippled line. (**F–O**) In situ hybridizations of spinal cord sections from control (F, G, K, L) and Tcf4ko (H, I, M, N) mice at E16.5 (F, H, K, M) and E18.5 (G, I, L, N) with antisense probes directed against *Mbp* (F–I) and *Plp1* (K–N), and corresponding quantifications (J, O). Scale bars: 100 μm. For quantifications absolute mean numbers of marker-positive cells ± SEM per thoracic spinal cord section of control (black bars) and Tcf4ko (white bars) mice are shown (*n* = 3–4 mice for each genotype, counting three separate sections). (**P**) Quantitative RT-PCR to determine *Nkx2.2, Myrf, Mbp, Plp1, Mag, Gjc2, Nfasc, Ank2, Acss2*,*Fa2h, Mboat1, Lss*,*Hsd17b7* transcript levels in the spinal cord of control and Tcf4ko mice at E18.5. Values for each gene in control spinal cord were set to 1 and levels in Tcf4ko spinal cord were set in relation to this (*n* = 3). Differences to controls were statistically significant as determined by two-tailed Student's *t* test (*P ≤ 0.05; **P ≤ 0.01; ***P ≤ 0.001).

Starting at E16.5, the first oligodendroglial cells enter the differentiation process in the thoracic spinal cord. This can be visualized by immunohistochemical detection of Myrf as the central transcriptional regulator of the myelination process (42). At E16.5, cells expressing Myrf protein are almost exclusively found in the spinal cord of control mice along the ventral midline (Figure 3A). At E18.5, occurrence of these cells has spread throughout the ventral marginal zone (Figure 3B). When age-matched Tcf4ko mice were analysed, Myrf protein was essentially absent in the spinal cord at E16.5 (Figure 3C). At E18.5, a few Myrf-positive cells appeared in the spinal cord of Tcf4ko mice, but their number was dramatically reduced compared to the control (Figure 3D). The strong reduction of Myrf-positive cells at both prenatal time points was confirmed in quantifications (Figure 3E).

Very similar results were also obtained when in situ hybridizations were performed on spinal cord sections of control and Tcf4ko mice at E16.5 and E18.5 for transcripts of the *Mbp* and *Plp1* genes (Figure 3F–O). The strong reduction of *Mbp* and *Plp1* transcript levels was also confirmed at E18.5 by quantitative RT-PCR on spinal cord tissue (Figure 3P). RT-PCR experiments additionally showed reduced expression for *Myrf* and for several other genes that are induced during oligodendroglial differentiation and code for structural components of the myelin sheath such as *Mag*, *Gjc2* (also known as Connexin-47), paranodal proteins such as *Nfasc* and *Ank2* or for enzymes involved in lipid biogenesis such as *Acss2*,*Fa2h, Mboat1, Lss* and *Hsd17b7* (Figure 3P). In contrast, transcript levels were comparable between control and Tcf4ko spinal cord tissue for *Nkx2.2*, a gene that starts to be expressed in pre-myelinating oligodendrocytes and is required for oligodendroglial differentiation (43). These results indicate that oligodendroglial cells in Tcf4ko mice get arrested in the pre-myelinating stage and that induction of terminal differentiation is strongly impaired.

### Effects of Tcf4 deletion on oligodendroglial development in slice culture

The peak of oligodendrocyte differentiation occurs during the first weeks after birth and cannot be studied in Tcf4ko mice because of their immediate postnatal death. To circumvent this problem we generated forebrain slices of newborn control and Tcf4ko mice and cultured them for up to 4 weeks *ex vivo* before immunohistochemical analysis. It has been shown that oligodendrocytes differentiate on schedule in these organotypic slice cultures (44).

**Figure 4.**
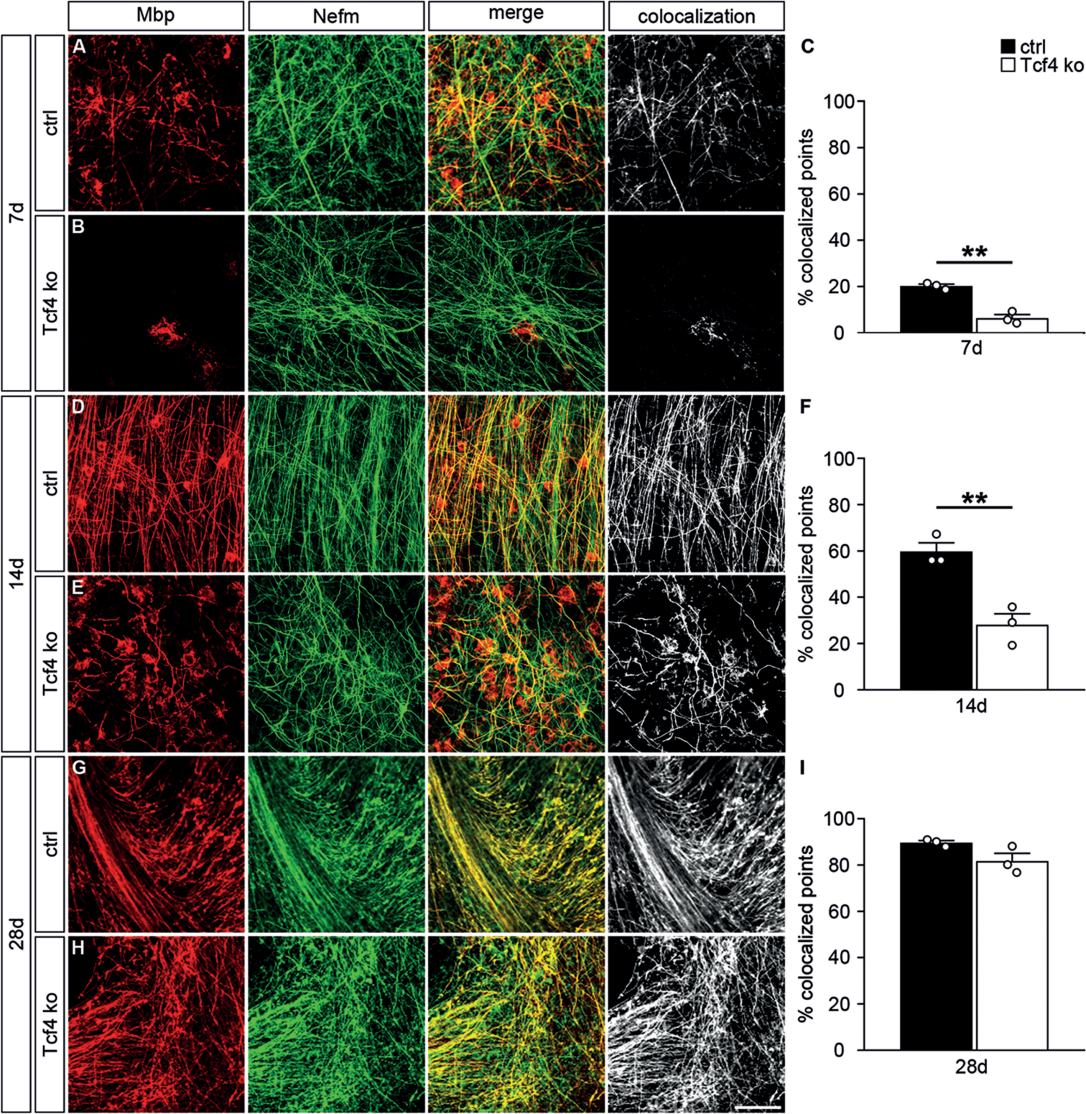
Impaired oligodendroglial differentiation in organotypic slice cultures of Tcf4ko mice. (**A, B, D, E, G, H**) Immunohistochemical stainings of organotypic slices from forebrain of control (ctrl) (A, D, G) and Tcf4ko (B, E, H) newborn mice with antibodies directed against Mbp (red) and Nefm (green) after seven days (7d) (A, B), 14 days (14d) (D, E) and 28 days (28d) (G, H) in culture. Single stainings are shown (two left panels) as well as their merge (third panel) and the colocalizing signal (white, right panel). Scale bar: 100 μm. (**C, F, I**) From these and similar stainings, quantifications were performed to determine the relative area with colocalized Mbp and Nefm signals using the Colocalization Threshold plugin of ImageJ (*n* = 3 slices from separate animals per genotype, counting at least 0.15 mm^2^ per slice). Differences to controls were statistically significant after 7 and 14 days in culture as determined by two-tailed Student's *t* test (**P ≤ 0.01).

After one week in culture, substantial immunoreactivity could be detected for Mbp in control slices (Figure 4A, left panel). A fraction of the Mbp signal aligned with Nefm (also known as NF165) as a marker for axons in white matter regions (Figure 4A, middle panels). Using colocalization of both markers as an approximation for the myelination process (Figure 4A, right panel), a substantial amount of myelin had been generated in control organotypic cultures during the first week (Figure 4C). In contrast, very little Mbp signal was detected in Tcf4ko slices and most of this signal did not colocalize with Nefm arguing that almost no myelination had occurred in organotypic slice cultures from Tcf4ko mice during the first week (Figure 4B, C).

After two weeks in culture, myelination had strongly proceeded in the controls with colocalization of Mbp and Nefm signal in 59 ± 4% of the analysed area (Figure 4D, F). Mbp was now also clearly detectable in the Tcf4ko slices (Figure 4E). The area in which Mbp and Nefm signal colocalized in Tcf4ko slices corresponded in extent roughly to the colocalized area in control slices one week earlier (compare Figure 4F with Figure 4C).

By 4 weeks, statistically significant differences were no longer detected between both genotypes in organotypic slice cultures regarding Mbp expression or amount of myelination (Figure 4G–I). We conclude from these findings that myelination is severely delayed in the absence of Tcf4, but eventually catches up.

### Effects of Tcf4 on development of cultured oligodendroglial cells

**Figure 5.**
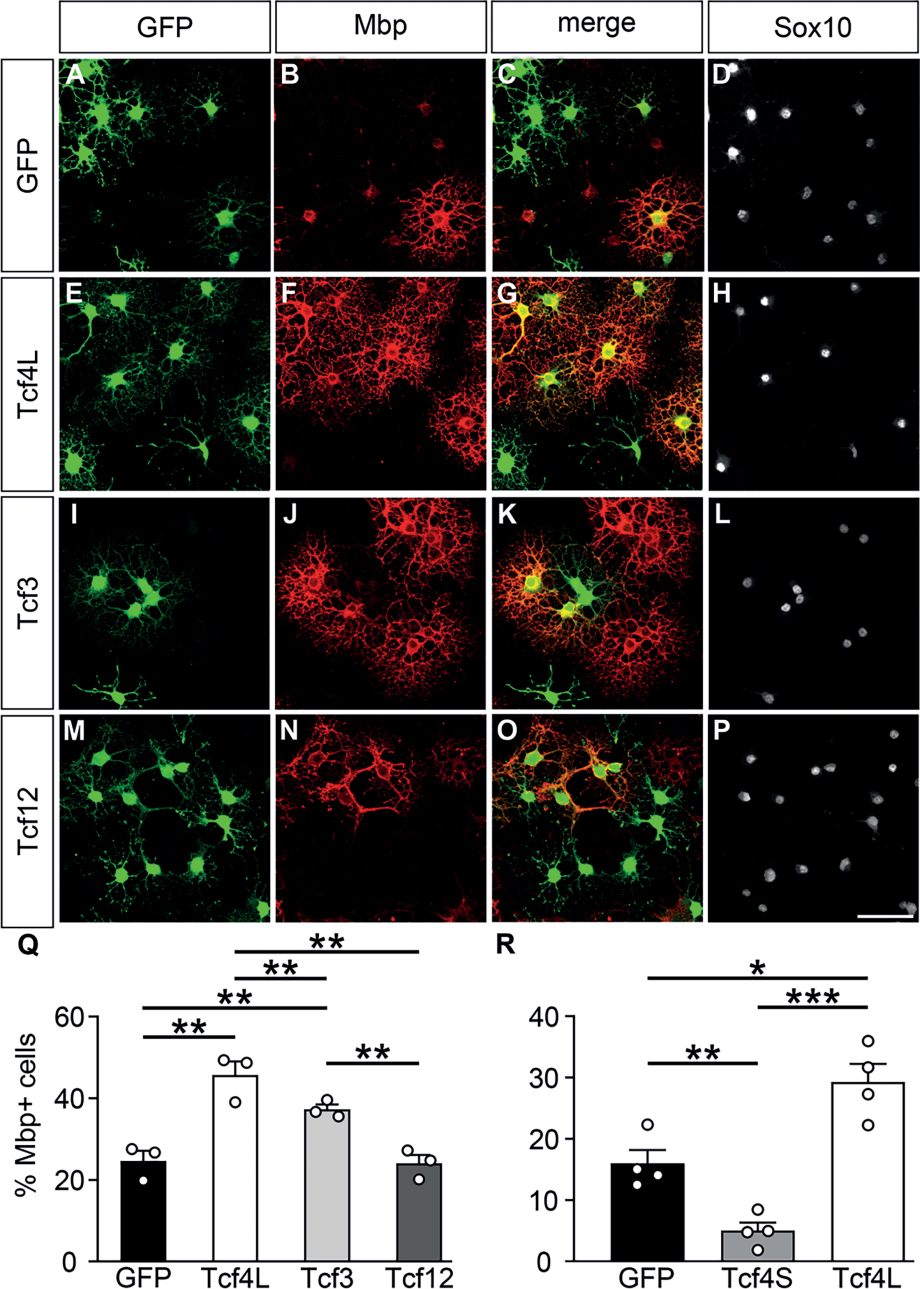
Influence of increased expression of class I bHLH proteins on oligodendrocyte differentiation *in vitro*. (**A–P**) Immunocytochemical stainings of rat oligodendroglial cells with antibodies directed against Gfp (green, A, E, I, M), Mbp (red, B, F, J, N) and Sox10 (white, D, H, L, P). Cells were kept for 6 days in differentiating conditions after transduction with control (A–D), Tcf4L- (E–H), Tcf3- (I–L) and Tcf-12 (M–P) expressing retrovirus. Single stainings are shown as well as their merge (C, G, K, O) as indicated above the panels. Scale bar: 50 μm. (**Q**) Quantifications were performed to determine the fraction of Mbp-expressing cells among all transduced cells (*n* = 3 cultures from separate transductions, counting at least four representative visual fields per transduction). (**R**) In a similar manner, quantifications were performed to determine the fraction of Mbp-expressing cells among cells transfected with expression plasmids for GFP alone or in combination with Tcf4L and Tcf4S isoforms (*n* = 4 separately transfected cultures, counting at least four representative visual fields per transfection). Differences to controls were statistically significant as determined by one way ANOVA with Bonferroni correction (*P ≤ 0.05; **P ≤ 0.01; ***P ≤ 0.001).

In Tcf4ko mice, the long isoform of this class I bHLH protein is missing in all cells. Therefore, it is not clear, whether the oligodendroglial differentiation defect in Tcf4ko mice is cell-autonomous or an indirect consequence of Tcf4 loss in other cell types. To differentiate between these possibilities we analysed the influence of increased Tcf4L levels on oligodendrocyte differentiation following retroviral transduction (Figure 5A–Q) or transfection (Figure 5R) of cultured rat oligodendroglial cells. When transduced with a GFP-expressing control virus, ∼25 ± 2% of Sox10-positive oligodendroglial cells had turned into Mbp-positive oligodendrocytes after six days under differentiating conditions (Figure 5A–D, Q). However, under identical conditions, ∼45 ± 4% had become Mbp-positive when transduced with a virus expressing Tcf4L (Figure 5E–H, Q). We conclude from this finding that Tcf4L promotes oligodendrocyte differentiation in a cell-autonomous manner. For transfected cells, the rate of Mbp-positive cells increased from 16 ± 3% for controls to 29 ± 3% for Tcf4L-transfected cells (Figure 5R). Intriguingly, Tcf4S-transfected cells exhibited even lower numbers of Mbp-positive cells than controls arguing that the differentiation promoting effect of Tcf4 is specific to the long isoform.

When transductions were carried out with retroviruses expressing Tcf3 (E47 isoform), the number of Mbp-expressing cells among transduced oligodendrocytes was again increased over control, although at a lower rate than after Tcf4L transduction (Figure 5I–L, Q). In contrast, the long variant of Tcf12 failed to increase the percentage of Mbp-expressing cells (Figure 5M–Q). This argues that Tcf4L and Tcf3 (but not Tcf12) have the capacity to promote oligodendroglial differentiation with different efficiencies.

**Figure 6.**
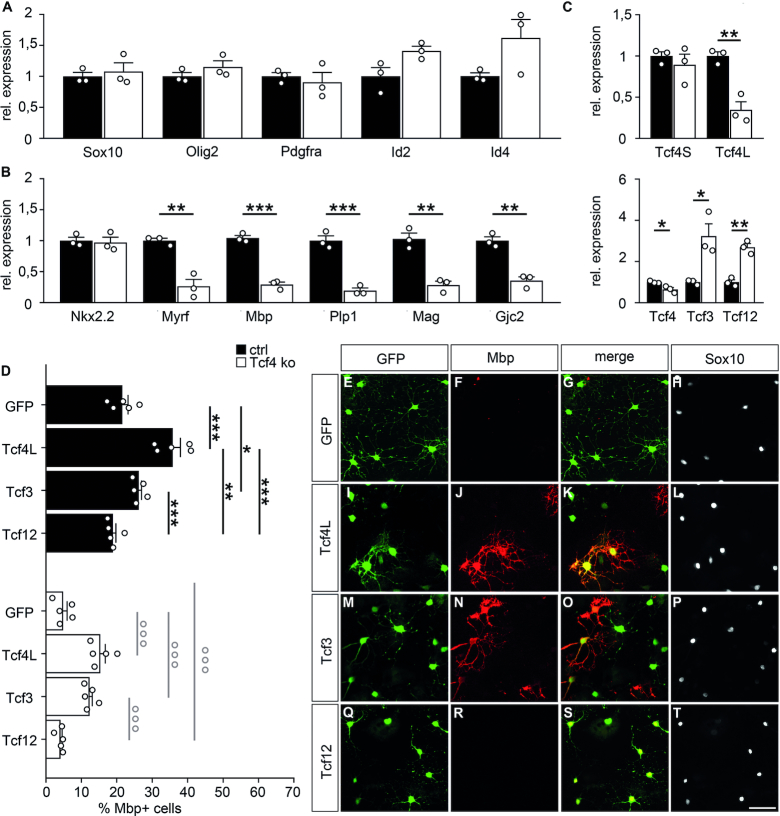
Differentiation of oligodendroglial cells from Tcf4ko mice in culture. (**A–C**) Quantitative RT-PCR to compare transcript levels for *Sox10, Olig2, Id2, Id4, Pdgfra*, (A), *Nkx2.2 Myrf, Mbp, Plp1, Mag, Gjc2* (B), *Tcf3, Tcf4, Tcf12* as well as the short (Tcf4S) and long (Tcf4L) isoforms of *Tcf4* (C) in oligodendroglial cells prepared from newborn control (ctrl, black bars) and Tcf4ko (white bars) mice and kept under differentiating conditions. Values for each gene in control oligodendroglial cells were set to 1 and levels in Tcf4ko cells were calculated in relation to this (*n* = 3). (**D**) Quantification of the fraction of Mbp-expressing cells among all transduced cells in oligodendroglial cultures from control and Tcf4ko mice (*n* = 5 cultures from separate transductions, counting at least three representative sections per slide). Differences to controls were statistically significant as determined by two-tailed Student's t test (A-C) or one way ANOVA with Bonferroni correction (D) (*P ≤ 0.05; **P ≤ 0.01; ***P ≤ 0.001). (**E–T**) Immunocytochemical stainings of Tcf4ko mouse oligodendroglial cells after transduction with Gfp- (E–H), Tcf4- (I–L), Tcf3- (M–P) and Tcf12- (Q–T) expressing retrovirus and six days culture in differentiating conditions using antibodies directed against Gfp (green, E, G, I, K, M, O, Q, S), Mbp (red, F, G, J, K, N, O, R, S) and Sox10 (white, H, L, P, T). Single stainings are shown as well as merged stainings as indicated above the panels. Scale bar: 50 μm.

In a second approach, we generated oligodendroglial cultures from newborn Tcf4ko mice and compared them to cultures from control mice. When kept under proliferating conditions, oligodendroglial cells from Tcf4ko mice exhibited *Sox10*, *Olig2*, *Id2, Id4*, *Pdgfra* and *Nkx2.2* transcript levels that were indistinguishable from controls (Figure 6A and B). In line with our mouse studies, this points to an unaltered oligodendroglial identity. However, after six days under differentiating conditions, transcript levels for *Myrf* and the myelin genes *Mbp*, *Plp1*, *Mag* and *Gjc2* were much lower in cultures from Tcf4ko mice than from controls confirming the cell-autonomous impact of Tcf4 on oligodendrocyte differentiation (Figure 6B).

Quantitative RT-PCR experiments confirmed the reduction of *Tcf4* levels in these cultures (Figure 6C). These changes were due to reductions in *Tcf4L*. In contrast, expression of the *Tcf4S* isoform was comparable to controls. Changes were, however, observed for both Tcf3 and Tcf12, which were expressed in increased levels in cultured oligodendroglial cells lacking Tcf4L (Figure 6C). This argues that there is a compensatory upregulation of Tcf3 and Tcf12 in the absence of Tcf4L in oligodendroglial cells. The fact that we have not seen this compensatory upregulation in total spinal cord RNA furthermore argues that this upregulation does not occur in other cell types of the developing spinal cord.

When analyzed at the single cell level by immunocytochemistry, control mouse oligodendrocytes differentiated with similar efficiencies as observed for rat oligodendrocytes (compare Figure 5Q to Figure 6D). Differentiation rates were further boosted by ectopic Tcf4L expression, and to a lesser extent by Tcf3 (Figure 6D). Ectopically expressed Tcf12 was again ineffective. When oligodendroglial cultures were prepared from Tcf4ko mice, very few cells converted into Mbp-positive oligodendrocytes after 6 days under differentiating conditions (Figure 6E–H). The differentiation rate was substantially lower than for control mouse oligodendroglial cells (Figure 6D). After reintroduction of Tcf4L by retroviral transduction, differentiation rates increased substantially, although it did not fully reach the rates determined for control oligodendroglial cells (Figure 6D, I–L). A less efficient rescue was also observed for Tcf3-transduced oligodendroglial cells (Figure 6D, M–P). By contrast, ectopic supply of Tcf12 had no effect on the number of Mbp-expressing cells (Figure 6D, Q–T). In summary, these results confirm the cell-autonomous effect of Tcf4L on oligodendrocyte differentiation and a partially redundant, less effective activity of Tcf3.

### Preferred physical interaction between Tcf4 and Olig2

Class I bHLH proteins frequently perform their function as heterodimers. In oligodendroglial cells, several class II bHLH proteins have been shown to contribute to the differentiation process, whereas class V and class VI bHLH proteins have rather been associated with maintenance of the proliferative state and anti-differentiation functions in OPCs (5). Considering the differentiation-promoting role of Tcf4, its function appeared more similar to those of known class II than class V and VI bHLH proteins in oligodendrocytes. Therefore, we concentrated on the oligodendroglial class II bHLH proteins Olig2, Olig1 and Ascl1 as possible interactors of Tcf4.

Using co-immunoprecipitations, we analysed the ability of Tcf4L to interact with Olig2, Olig1 and Ascl1, and compared it with the two other class I bHLH proteins. For that purpose, all proteins were overexpressed in transfected Hek293 cells. For lack of good commercially available antibodies, all class I bHLH proteins were tagged with an aminoterminal myc epitope, whereas Olig1 and Ascl1 were tagged with an aminoterminal T7 epitope.

**Figure 7.**
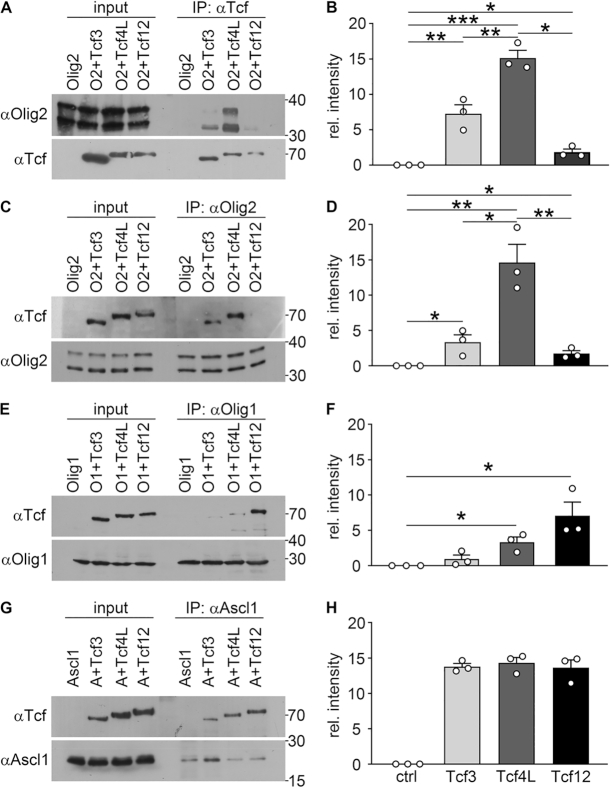
Physical interaction of oligodendroglial class I and class II bHLH proteins. (**A, C, E, G**) Co-immunoprecipitations were performed on extracts from transfected Hek293 cells that contained Olig2 (O2 in A, C), T7-tagged Olig1 (O1 in E) or T7-tagged Ascl1 (A in G) alone or in combination with a specific myc-tagged class I bHLH protein (Tcf3, Tcf4L or Tcf12) as indicated above the lanes. Antibodies for immunoprecipitations were directed against the myc tag (αTcf, A), Olig2 (αOlig2, C) or the T7 tag (αOlig1 in E, αAscl1 in G) as indicated at the top of each panel. The exact proteins present in each extract before (input) and after immunoprecipitation (IP) were visualized by western blotting using the antibodies listed on the left side of the panels. The precipitated protein is always shown in the lower panels, the co-precipitated in the upper ones. Numbers on the right indicate molecular weight markers in kDa. (**B, D, F, H**) On these and similar Western blots, densitometry was performed to determine the amount of co-precipitated protein after normalization to the amount of precipitated protein in each lane as relative intensity (*n* = 3 independent experiments). Differences in the amounts of co-precipitated proteins were statistically significant as determined by one-way Anova with Bonferroni correction (*P ≤ 0.05; **P ≤ 0.01; ***P ≤ 0.001).

In the first set of experiments, extracts were used that contained only Olig2 or Olig2 in combination with one of the class I bHLH proteins. When immunoprecipitations were carried out with an antibody directed against the myc tag present in the class I bHLH proteins, we detected substantial amounts of Olig2 in the precipitate, when Tcf4L was present (Figure 7A). Less Olig2 was precipitated when Tcf3 was present instead of Tcf4L and co-immunoprecipitation failed in the presence of Tcf12 or in the absence of a tagged class I bHLH protein. Quantification of three independent co-immunoprecipitations confirmed that Tcf4L is the preferential interaction partner of Olig2, followed by Tcf3 (Figure 7B). To confirm our results we used the same extracts to perform immunoprecipitatons with antibodies directed against Olig2 and probed the precipitate for the presence of class I bHLH proteins using antibodies directed against the myc epitope. In agreement with our previous experiments we detected much more Tcf4L in the precipitate than Tcf3 and little Tcf12 (Figure 7C, D). Intriguingly, the strength of Olig2 interaction correlates with the ability of each class I bHLH protein to promote oligodendrocyte differentiation, suggesting a causal link between both.

In a second set of experiments, we replaced Olig2 by Olig1 and repeated the immunoprecipitations with a T7-tag antibody. In this case, Tcf12 was preferentially co-immunoprecipitated, followed by Tcf4L and Tcf3 (Figure 7E). This preference again held up over three separate experiments (Figure 7F). In contrast to the clear preference of Olig1 and Olig2 for a specific class I bHLH protein, Ascl1 appeared to bind equally well to Tcf3, Tcf4L and Tcf12 as judged by corresponding co-immunoprecipitations (Figure 7G, H). This argues that Ascl1 is less selective than Olig2 or Olig1 with regards to its class I bHLH interaction partner.

### Genetic interaction of Tcf4 and Olig2 in mice

Considering the preferential interaction of Olig2 with Tcf4, we postulated that at least part of the oligodendroglial differentiation defect observed in Tcf4ko mice may be caused by disturbance of this interaction. Therefore, we generated compound mutant mice with heterozygous loss of Olig2 and heterozygous inactivation of Tcf4 (OT double het) and compared oligodendrocyte development in these double hets to single Tcf4 and Olig2 heterozygous mice (Tcf4 het and Olig2 het) as well as wildtype controls.

**Figure 8.**
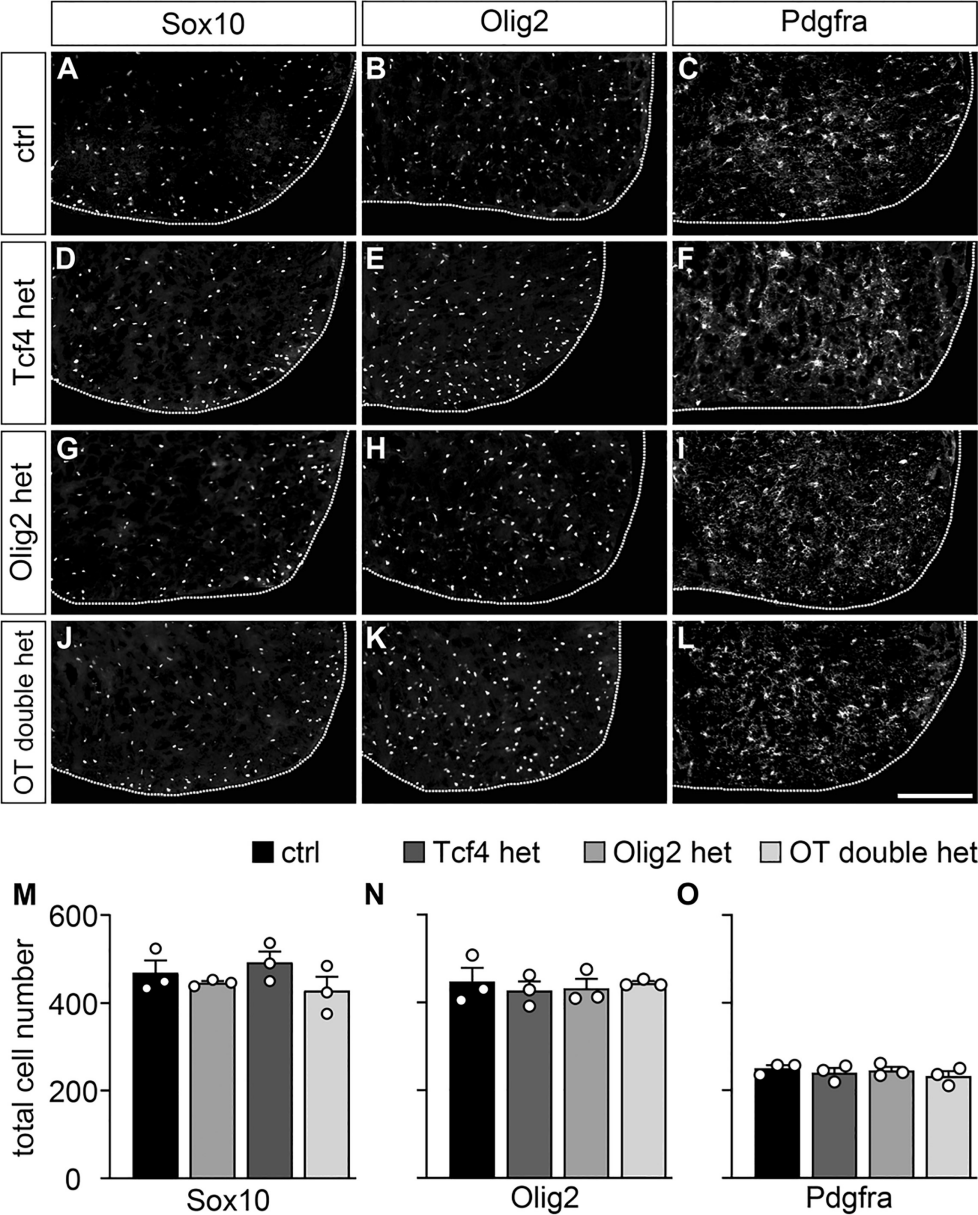
Normal lineage progression of oligodendroglial cells in OT double het mice. (**A–L**) Immunohistochemical stainings of spinal cord sections from wildtype control (ctrl, A–C), Tcf4 het (D–F), Olig2 het (G–I) and OT double het (J–L) mice at E18.5 with antibodies directed against Sox10 (A, D, G, J), Olig2 (B, E, H, K) and Pdgfra (C, F, I, L). For stainings, the right ventral horn is shown, placed on a black background and demarcated by a stippled line. Scale bar: 100 μm. (**M–O**) From these and similar immunohistochemical stainings, quantifications were performed to determine the absolute mean number of marker-positive cells ± SEM per thoracic spinal cord section (*n* = 3 mice for each genotype, counting three separate sections). No statistically significant differences to controls were detected.

**Figure 9.**
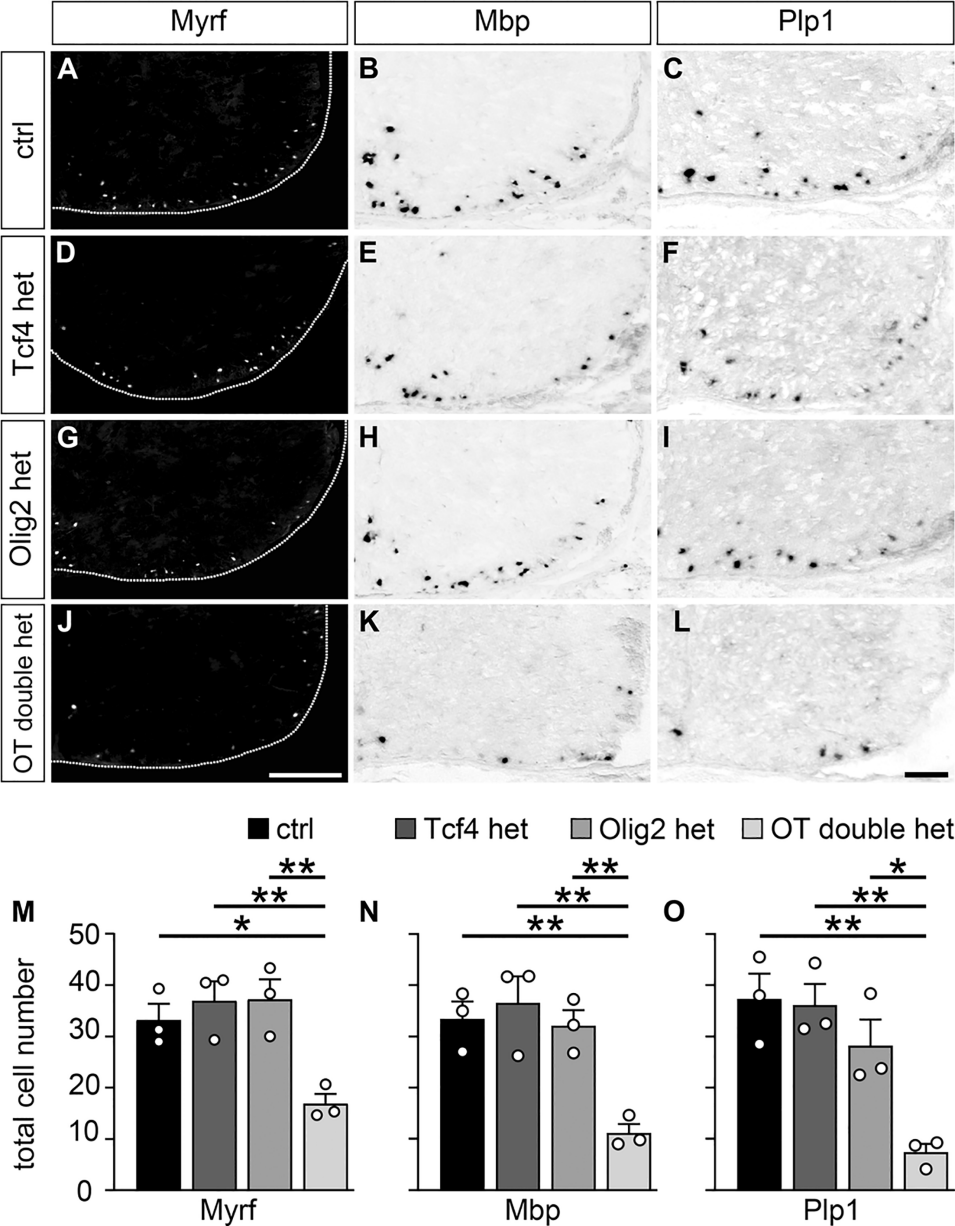
Impaired oligodendroglial differentiation in OT double het mice. (**A–L**) Immunohistochemical stainings with antibodies directed against Myrf (A, D, G, J) and *in situ* hybridizations with probes specific for *Mbp* (B, E, H, K) and *Plp1* (C, F, I, L) were carried out on spinal cord sections from wildtype control (ctrl, A–C), Tcf4 het (D–F), Olig2 het (G–I) and OT double het (J–L) mice at E18.5. The right ventral horn is shown. For immunohistochemical stainings, the spinal cord was placed on a black background and demarcated by a stippled line. Scale bar: 100 μm. (**M–O**) From these and similar experiments, quantifications were performed to determine the absolute mean number of marker-positive cells ± SEM per thoracic spinal cord section (*n* = 3 mice for each genotype, counting three separate sections). Differences to controls were statistically significant as determined by one way ANOVA with Bonferroni correction (*P ≤ 0.05; **P ≤ 0.01).

At E18.5, number and localization of oligodendroglial cells was completely identical among all genotypes when analysed with Sox10 or Olig2 as pan-oligodendroglial markers (Figure 8A, B, D, E, G, H, J, K, M, N). Similarly, no difference was detected for Pdgfra as OPC marker (Figure 8C, F, I, L, O). However, when numbers of differentiating oligodendrocytes were counted using either Myrf, *Mbp* or *Plp1* as marker, significant reductions were observed in OT double het mice, but not in Tcf4 het or Olig2 het mice (compare Figure 9A–I to Figure 9J–L). The observed oligodendroglial differentiation defect in OT double het mice was milder than in Tcf4ko mice and was no longer apparent after the first postnatal week (compare Figure 9M–O to Figure 3E, J, O). Nevertheless, it strongly supports the notion of a genetic interaction of Tcf4 and Olig2 during oligodendroglial differentiation.

### Joint activation of oligodendroglial regulatory regions by Tcf4 and Olig2

**Figure 10.**
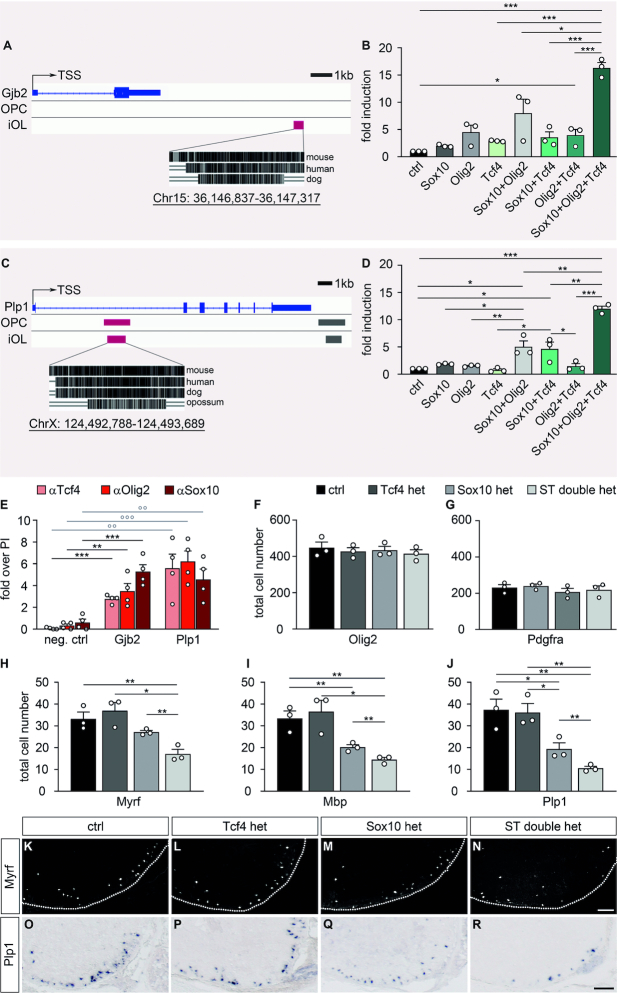
Joint influence of Tcf4, Olig2 and Sox10 on oligodendroglial gene expression. (**A, C**) Localization of evolutionary conserved regulatory regions with Olig2 binding capacity (red box) in OPCs or immature oligodendrocytes (iOL) in the *Gjb2* (A) and *Plp1* (C) genomic region relative to transcriptional start site (TSS) and exons (blue boxes). The exact genomic position according to rn4 is given at the bottom. (**B, D**) Activation of luciferase reporters under control of these regulatory regions from the *Gjb2* (B) and *Plp1* (D) genes in transiently transfected N2a cells by Olig2, Tcf4L, Sox10 and combinations thereof as indicated below the bars (n = 3; presented as fold inductions ± SEM, transfections without added transcription factors set to 1 for each regulatory region). (**E**) Chromatin immunoprecipitation on cultured differentiating oligodendroglial cells with antibodies directed against Tcf4, Olig2 and Sox10. Enrichment of *Gjb2*, *Plp1* and control (neg. ctrl) genomic region in the immunoprecipitates was determined relative to preimmune/IgG controls (PI) that were arbitrarily set to 1. Experiments were performed four times with each PCR in triplicate. (**F–J**) Quantification of the number ± SEM of Olig2-positive oligodendroglial cells (F), Pdgfra-positive OPCs (G), Myrf-, *Mbp*- or *Plp1*-positive oligodendrocytes (H–J) per thoracic spinal cord section in wildtype control (ctrl), Tcf4 het, Sox10 het and ST double het mice at E18.5 (n = 3 mice for each genotype, counting three separate sections). (**K–R**) Immunohistochemical stainings with antibodies directed against Myrf (K-N) and in situ hybridizations with a probe directed against *Plp1* (O–R) on spinal cord sections from wildtype (ctrl; K, O), Tcf4 het (L, P), Sox10 het (M, Q) and ST double het (N, R) mice at E18.5. The right ventral horn is shown. For immunohistochemical stainings, the spinal cord was placed on a black background and demarcated by a stippled line. Scale bar: 100 μm. Statistical significance was determined by one way ANOVA with Bonferroni correction (*P ≤ 0.05; **P ≤ 0.01; ***P ≤ 0.001).

To further confirm the potential interaction of Tcf4 and Olig2, we searched for evidence of joint activation of genes with relevance for oligodendrocyte differentiation by both factors. For this purpose, we screened publicly accessible ChIP-Seq data to identify evolutionary conserved regions with Olig2 binding capacity in the vicinity of *Myrf, Mbp, Plp1, Mog, Mobp, Gjb1* (Connexin-32) and *Gjb2* (Connexin-26) as genes expressed in differentiating oligodendrocytes (45). Identified regions were inserted into luciferase reporter plasmids. When transfected into N2a cells, most failed to be stimulated in reporter gene assays by Olig2 and/or Tcf4 independent of whether Sox10 as a general activator of myelin genes was present or not (29,46). However, an evolutionary conserved regulatory region 12 kb downstream of the transcriptional start site of the *Gjb2* gene and another one in intron 1 of the *Plp1* gene 5kb downstream of the transcriptional start site exhibited noteworthy responses (Figure 10A, C). Both regions were at best weakly activated by Sox10, Olig2 or Tcf4 alone (Figure 10B, D). Combinations of two transcription factors also led to a modest upregulation at best. Substantial rates of activation were only observed in the presence of Olig2, Tcf4 and Sox10 arguing that these regions can only be activated by this specific combination of transcription factors (Figure 10B, D). Chromatin immunoprecipitation from differentiating oligodendroglial cells with antibodies directed against Tcf4, Olig2 and Sox10 confirmed *in vivo* enrichment of all three transcription factors on the regulatory regions from the *Gjb2* and *Plp1* genes (Figure 10E). This was not observed for an unrelated genomic control region.

These results confirm a physiologically relevant interaction between Tcf4 and Olig2, but also suggest a further link to Sox10. To probe the relationship between Tcf4 and Sox10 on a genetic level, we analysed compound mouse mutants with heterozygous Sox10 loss and additional heterozygous Tcf4 inactivation (ST double hets). As previously observed for OT double hets, we did not observe differences between ST double hets, the respective single heterozyogus mice (Tcf4 hets and Sox10 hets) and wildtype controls in overall oligodendroglial numbers or numbers of Pdgfra-positive OPCs at E18.5 (Figure 10F, G). In contrast to other mice with single heterozygous gene alterations, Sox10 hets already exhibited a decreased number of Myrf-, *Mbp*- and *Plp1*-positive differentiating oligodendrocytes (Figure 10H–M, O–Q). This decrease was even bigger in the ST double hets (Figure 10H–J, N, R), supporting the existence a genetic interaction between Tcf4 and Sox10.

## DISCUSSION

In this manuscript, we show that Tcf4 is required for proper terminal differentiation of oligodendrocytes and CNS myelination *in vivo* and *ex vivo*. We furthermore provide evidence from both gain-of-function and loss-of-function studies that the effect of Tcf4 is in large parts cell-autonomous. Data from organotypic slice cultures indicate that the effect of Tcf4 is substantial as myelination appears to be severely delayed.

We had not expected to see such a dramatic effect on oligodendroglial development, as we only deleted the long isoform of Tcf4. In addition, oligodendroglial cells also express the other two class I bHLH paralogs Tcf3 and Tcf12, which are believed to be highly similar and redundant in function to Tcf4 as evident from their roles in lymphocyte development and the ability of Tcf12 to replace Tcf3 in vivo (41,47). Considering that all three are expressed at high levels in developing oligodendrocytes and that *Tcf3* and *Tcf12* transcript levels are upregulated in the absence of the long Tcf4 isoform, we would have predicted compensation by the remaining Tcf3 and Tcf12. The most parsimonious explanation for our results therefore is that the long isoform of Tcf4 has unique properties that cannot be compensated for during oligodendrocyte differentiation by the short Tcf4 isoform or the Tcf3 and Tcf12 paralogs. Such an assumption is supported by our finding of significant differences between Tcf4 isoforms and among class I bHLH proteins in their ability to promote oligodendrocyte differentiation or rescue the differentiation defect in Tcf4-deficient oligodendroglial cells in culture.

Class I bHLH proteins are capable of homo- as well as heterodimerization. Considering that class II bHLH proteins act primarily as heterodimers with a class I bHLH protein (4), heterodimerization appears particularly relevant for class II bHLH function. Heterodimerizing class II bHLH proteins include many of the proneural factors that drive neurogenesis in the early nervous system such as Atoh1 and the neurogenins. In fact, Atoh1 has been shown to rely specifically on Tcf4 for its function during CNS development (48). However, heterodimerization of class I bHLH proteins is not confined to class II bHLH proteins. Heterodimerization partners also include class V and class VI bHLH proteins.

In the context of oligodendroglial development, important functions have been described for the class II proteins Olig2, Olig1 and Ascl1, the class V proteins Id2 and Id4 and the class VI protein Hes5 (5). The Id proteins and Hes5 have been primarily associated with functions in OPCs where they maintain precursor state, promote proliferation and prevent differentiation (10–12). Considering that Tcf4 rather functions as a differentiation promoting factor, it appeared unlikely that its interactions with class V or class VI bHLH proteins are responsible for the observed effects on oligodendrocyte differentiation. Heterodimerization with class II bHLH factors was much more likely of relevance, as all three oligodendroglial class II proteins promote oligodendrocyte differentiation and myelination (6,8,9).

In this context, it is of particular interest that we detected a clear bias of Olig2 for Tcf4, whereas Olig1 chose Tcf12 as its preferred interaction partner. Ascl1, in contrast, appeared to heterodimerize equally well with all three class I bHLH proteins. Considering this special link between Olig2 and Tcf4, it therefore seemed likely that Tcf4 exerts at least part of its role in oligodendroglial differentiation as a heterodimer with Olig2. In support of such an assumption, oligodendroglial differentiation was impaired in compound mutant mice with heterozygous inactivation of *Olig2* and *Tcf4*, but normal in single heterozygous mice. This finding proves the existence of a genetic interaction between Tcf4 and Olig2.

Oligodendroglial cells from Tcf4ko mice eventually overcome their differentiation and myelination problems. This argues that Olig2-Tcf4 heterodimers can be functionally replaced by other heterodimeric species that contain Olig2 or Olig1, a close relative with at least partially redundant function in oligodendroglial differentiation (49). From our data it seems plausible to assume that the active species that eventually replace Olig2-Tcf4 heterodimers are Olig2-Tcf3 and/or Olig1-Tcf12.

Olig2-Tcf4 heterodimers are probably not the only context, in which Tcf4 functions in oligodendrocytes. Formation of heterodimers with other class II bHLH proteins such as Ascl1 may be relevant as well. Additionally, Tcf4 may be involved in further functionally important interactions with non-bHLH transcription factors such as Sox10. In support of such an assumption, we found evidence that some regulatory regions from myelin genes are not only occupied by Tcf4 and Olig2 but also by Sox10, and respond in reporter gene assays only to a combination of all three transcription factors. Mouse genetics furthermore provide evidence for a genetic interaction between Tcf4 and Sox10 in oligodendrocytes. Efficient activation of the oligodendroglial differentiation program may therefore require interactions of Tcf4 with both Olig2 (probably as a heterodimer) and Sox10. The exact molecular nature of this ménage à trois and the possible existence of complexes that contain all three proteins will have to be investigated in future studies.

Inactivations, deletions and single nucleotide variations in human TCF4 have been associated with syndromic and non-syndromic forms of intellectual disability, schizophrenia, autism and several nervous system dysfunctions such as breathing abnormalities and seizures in Pitt-Hopkins syndrome (22). Features of autistic or schizophrenic behavior such as deficits in social interaction, ultrasonic vocalization, and associative learning have also been observed in mice with Tcf4 haploinsufficiency (50). It is commonly assumed that the observed deficits and dysfunctions are caused by Tcf4-dependent alterations in neurons. Our finding of a role of Tcf4 in oligodendroglial differentiation and myelination at least opens the possibility that part of the functional deficits in patients and affected persons are also caused by altered properties of oligodendrocytes and the impact of these alterations on neuronal circuit function. Future experiments will have to look into this possibility.

## DATA AVAILABILITY

All data generated or analysed during this study are included in this article.
